# Knowledge, attitude, practice and perceived barriers of natural disaster preparedness among Nepalese immigrants residing in Japan

**DOI:** 10.1186/s12889-022-12844-3

**Published:** 2022-03-12

**Authors:** Aliza K. C. Bhandari, Osamu Takahashi

**Affiliations:** Graduate School of Public Health, St. Luke’s International University, Tokyo, Japan

**Keywords:** Disaster preparedness, Immigrants, Nepalese, Natural disaster, Japan

## Abstract

**Background:**

Natural disasters have increased during the last several decades all over the world. Due to its geographical and climate conditions, Japan has long been vulnerable to several natural disasters. Coping with disasters is a major challenge overall and even harder for foreigners residing in Japan. Thus, the objective of this study was to examine the perceived knowledge, attitude, practice and perceived barriers of disaster preparedness among Nepalese immigrants in Japan.

**Methods:**

A cross-sectional study was conducted among Nepalese immigrants residing in Japan with an online survey questionnaire. The questionnaire was validated and then administered. The participants were recruited via Facebook for this survey. Bivariable and multivariable logistic regression analyses were conducted to examine the factors associated with the perceived knowledge, attitude and practice of Nepalese immigrants regarding disaster preparedness.

**Results:**

A total of 404 respondents were analyzed in this study and among them two-third were male. We found that the mean score of disaster preparedness practice was lowest than the knowledge and attitude (mean $\pm SD$ = 15.86 $\pm$ 5.52) as evidenced by the majority of the participants not being prepared for disaster situations and a limited proportion had ever taken necessary natural disaster preparedness measures. Japanese language was identified as the major barrier in assessing the knowledge, attitude and practice regarding disaster preparedness and was significantly associated with the knowledge level of disaster preparedness after adjusting for some socio-demographic covariates. (aOR: 1.84, 95% CI: (1.04 – 3.25)).

**Conclusions:**

This study observed that the perceived knowledge and practices regarding natural disasters are very poor while barriers to access these are substantial among Nepalese immigrants in Japan. As Japanese language was identified as a major barrier, the availability of language translation services in every health care sector also in the government offices of Japan might encourage people to learn more about disaster preparedness.

**Supplementary Information:**

The online version contains supplementary material available at 10.1186/s12889-022-12844-3.

## Background

According to the International Federation of Red Cross Society (IFRCS) disaster are any unforeseen events leading to the dysfunction of our society resulting in economic, materialistic and human losses to the extreme limit where the society can no longer handle the adverse effects [1]. Disasters mostly have natural origin; however, man-made disasters like occupational hazards, infectious disease outbreak, terrorist attacks and others are also on the rise [2, 3]. Over the last few decades, the rate of natural disaster occurrences have increased substantially with a visible rise of about 80% being observed during 1980–2009. The Emergency Events Database (EED) identified nearly 7000 natural disasters globally between 1994 and 2013 which claimed 1.35 million lives and have affected an additional hundreds of millions of people. In the subsequent years, nearly 8,000 people lost their lives due to various forms of natural disasters [4]. Several studies emphasize Asia to be the region with highest number of disaster occurrence [5–7]. Japan, often called as the country of volcanoes, has been vulnerable various natural disasters such as earthquakes, volcanic eruptions, tsunamis, typhoons, wildfires and heavy snow falls. In 2019 alone Japan was hit by several natural disasters with typhoon and heavy rain being the most frequent disasters of the year [8]. According to the white paper on disaster management in Japan, nearly half of the fatalities worldwide caused by natural disasters are concentrated in the low-income countries like Nepal and more than one-fourth of those fatalities are concentrated in the lower-middle-income countries [9]. It is interesting to note that more than 17% of total damages that occurred worldwide due to natural disasters between 1984 and 2013 occurred in Japan [9].

Nepal, on the other hand, due to its topography and climatic condition is also considered as one of the most disaster-prone countries where occasional disasters like landslides, floods, earthquakes, fire, thunderbolts, etc. occur each year leading to substantial health morbidity and mortality [10]. One of the major earthquake disasters in 2015 shook the country with around 9,000 deaths, thousands of injuries and a large population becoming homeless due to the destruction of their homes. It was estimated that this single disaster left the country with damage of about 10 billion US dollars (USD) which was nearly half of Nepal’s nominal GDP [11]. The earthquake disaster of 2015 helped mirror the country’s disaster management policies and their effectiveness. In contrary to Japan where the government has strengthened the laws and policies regarding disaster management every year, the Nepal government still lacks a rigid control on disaster management due to the unstable political situation [12, 13]. Meanwhile, not only the country’s policies but several readiness programs conducted in the communities of similar low-income countries are primarily focused on the emergency response and health care professionals and not on the individual preparedness levels of the general population. This might have left the public unprepared for such emergency event response further adding to their risk profile [14].

Natural disasters are unpredictable and unavoidable but their effects could be minimized by preparedness related knowledge and practice [15]. Japan is home to nearly a hundred thousand Nepalese immigrants [16]. Being from a vulnerable country like Nepal with low health literacy and different cultural beliefs and attitudes than the Japanese population, Nepalese immigrants might be prone to suffer more during disaster situations [17]. Several studies conducted in Japan have already shown the low health care accessibility of Nepalese in Japan due to the cultural and language barriers [18]. Japan has its own disaster management protocol like other parts of the world which has been effective in preventing morbidity and mortality in the past [19]. However, its effectiveness towards saving the lives of foreign residents has not yet been examined yet as most of the information provided by the Japanese government related to disaster preparedness and management is either in the Japanese or English languages. There are limited number of official government websites providing information in multiple languages, and the Nepalese language is rarely included among those language preferences [20]. Nepalese coming to Japan are mostly students generally those with just a high school degree and blue-collar workers who rarely could understand English. For such population learning or understanding a third language like Japanese is more complex [21, 22].

There is enough evidence that disaster preparedness is crucial to mitigate the probable effects of natural disasters in high-risk population [23, 24]. Several studies have identified factors such as the previous experience with the disaster situations, the income level, area of residence, occupation and other demographic factors to be associated with the disaster preparedness knowledge of individuals [25]. However, there is a dearth of studies focusing on the disaster preparedness knowledge, attitude and practice of immigrants living in Japan. Thus, this study hypothesizes that there are specific socio-demographic factors might be hindering the perceived knowledge, attitude and practice of Nepalese immigrants living in Japan regarding natural disasters, and there are barriers to acquiring adequate information for enhancing their knowledge, attitude and practice regarding disaster preparedness.

## Methodology

### Study design and eligibility of participants

A cross sectional study was conducted among Nepalese immigrants who are residing in any prefecture of Japan with more than three months stay in Japan and who provided written informed consent. Immigrants less than 18 years old (minors) and those who did not respond about their visa status (residency status) or those who had tourist visa status were excluded from the study.

### Data collection tool

#### Instrument design

We designed the questionnaire using three-step procedures: literature review, content generation through expert’s opinion, focus group discussion and factor analysis followed by pre-testing. At the first step, we gathered information related to the natural disaster by conducting in-depth literature review and identified some validated questionnaires which were related to natural disaster preparedness [25–27]. Next, we abstracted relevant information from these documents and generated items that were relevant to the research question and drafted a 63 items questionnaire. We refined and organized the items within the questionnaire with the help of an expert panel consisting of seven members, followed by a focus group discussion with six members from the representative population. This procedure was followed by factor analysis and Cronbach alpha calculations. The finalized items were translated into Nepali language by the author who is a native Nepali speaker (AKCB). Further, back translation of the Nepali version of the questionnaire was done by an expert. We requested two other language experts to check both Nepali and English versions of the questionnaire for any discrepancies, and the Nepali version of the questionnaire was finalized after amending the suggestions from the language experts [28, 29].

To explore the knowledge level of the participants we asked them to rate their knowledge on various natural disasters based on a 5-point Likert scale ranging from “very less” to “very high” level of knowledge. There were nine items to assess knowledge level of various natural disasters; hence, the score ranged from a minimum of 0 to a maximum of 40. Similarly, to assess their attitude towards various forms of natural disasters, we requested them to rate their concern on various disasters also using a 5-point Likert scale ranging from “Not concerned at all” to “Highly concerned” with same number of items as in the knowledge section providing a cumulative score from 0 to 40. Meanwhile, the practice of immigrants was assessed in two steps. At first we asked them their willingness to prepare themselves for the natural disaster situations like their assertiveness in gathering information regarding disasters, willingness to participate in a disaster preparedness training, etc. using a 5-point Likert scale ranging from “Don’t want to do” to “Have already done” however, “Don’t want to do” had a value of 0. Hence, the score for the assertiveness in practice related to disaster preparedness ranged from 0 to 40. Lastly, we asked them some straight forward questions regarding their current disaster preparedness practices including their food and water storage, preparation of emergency bag, their emergency exit or evacuation plans and so on in a 4-point Likert scale ranging from “No” to “Not needed”. Here “Not needed” had a value of 0, hence, we had a score for practice ranging from 0 to 39.

#### Dissemination of survey questionnaire

The questionnaire was distributed using an online survey software called “QuestionPro”, an online survey software from which we can create our survey questionnaire and use its link to distribute it among desired participants. QuestionPro software has advanced features to detect multiple responses by tracking the IP address, the country of filling of the respondents and it can also provide information on time taken by an individual to complete the questionnaire. Participants were informed about the survey via social networking sites, Facebook pages of major organizations working for Nepalese immigrants in Japan such as NRNA (Non-Residential Nepalese Association), Nepali helping hands and via some Nepali online media such as “Nepalnewspost” and “Japansamachar”. The link to the survey consent form and the questionnaire was also distributed via similar networking sites in between January and March 2021.

#### Validation of the questionnaire

We sent the first draft of the questionnaire with 63 items to a panel of 7 experts who had achieved academic excellence or are working in the disaster management field. Experts were all Nepalese residing in various parts of the world, one from the USA, two from Nepal and four from Japan. We asked these experts to label each item of the questionnaire into any of these three categories: “not necessary”, “useful but not essential” or “essential”. We asked experts to label the item as “not necessary” if they thought it was not practically applicable to Nepali immigrant society or was something that might be socially and politically inappropriate. Similarly, they were instructed to label an item under “useful but not essential” if the item could be merged with other components or might not be needed to meet the research objectives. Meanwhile, they were asked to label an item as “essential” when they felt that the item was important to fulfill the objectives of this research and was highly relevant to the context of Nepali immigrants residing in Japan [30]. We calculated the content validity ratio (CVR) for each item. A total of 8-items were excluded as CVR was less than 0.50 and 2-items were added based on the suggestions of our experts and revisions were made on three other items.

As a next step, we conducted a focus-group discussion (FGD) with 6 Nepalese immigrants residing in Japan (3 females and 3 males, aged between 24 and 50 years). These FGD members were asked about the relevancy and complexity of each items of the questionnaire and were requested to suggest any changes that might be necessary. According to the response from them, we excluded four more items and modified three other items so that general Nepalese immigrants could understand the purpose of the survey. After conducting these changes, the questionnaire was discussed among the authors (AKCB, OT), few more modifications and revisions were made resulting in a 48-item questionnaire. This 48-itemed questionnaire was sent back to the experts with a request to check the content validity index (CVI) using a 4-point Likert scale: “Highly relevant”, “Quite relevant”, “Somewhat relevant” and “Not relevant”. We used the following formula to calculate item level CVI (I-CVI) and the scale level CVI (S-CVI) [30, 31]. All of the items had score between 80 to 100%; hence no items were omitted. We also calculated the modified Kappa statistic using the following formula:

$$\mathrm K=\left(\mathrm I-\mathrm{CVI}-\mathrm{Pc}\right)/\left(1-\mathrm{Pc}\right).$$

Where, P_C_ is the probability of chance agreement, calculated for each item by the following formula:

$$\mathrm{PC}=\left[\mathrm N!/\mathrm A!\left(\mathrm N-\mathrm A\right)!\right]\ast.5^{\mathrm N}.$$

In this formula, N is the number of experts, A is the number of experts who agreed that the item was relevant.

All items with a kappa value ≥ 0.74 were considered excellent and hence all 48-items were included in the final questionnaire. In addition to this, we added a simple multiple-choice question to identify the inattentive responses, such as “In which planet do you live in?”; participants who responded to this question incorrectly were excluded from this study.

#### Reliability of the questionnaire

Pre-testing of the questionnaire was conducted on 10% of the sample and a response rate of over 85% was obtained. Cronbach alpha was calculated using Stata to find the inter-item correlations for all pairs of variables. The value of Cronbach alpha was 0.88 which indicated good reliability of the instrument [32].

#### Sample size calculation

Total number of Nepalese immigrants in Japan (N) was 92,804 as of June, 2019.

The following formula was used to calculate the sample size:

$$n = Z^{2} P\left( {1 - P} \right) \div d^{2}$$

where, *n* = sample size.

*Z* = Z statistic for a level of confidence.

*P* = expected prevalence or proportion of knowledge on disaster preparedness.

*d* = precision.

A non-probability purposive sampling technique was used in selecting the samples. We used the above-mentioned Daniel formula to estimate the minimum sample size for the study. Where (p) is a value of expected proportion considered as 50%, (z) is the statistic for 95% confidence interval, (d) is an error of deviation of 5%. The calculated minimum sample size was 384 [33, 34].

### Statistical analysis

The overall knowledge and attitude were categorized into “good” and “bad” using Modified Bloom’s cut-off point; “good” if the score was above 60% (score more than 24) otherwise categorized as “poor”. Similarly, Bloom’s cut-off point of ≥ 80% (score ≥ 32) was categorized as “good practice” and score < 80% as “poor practice” [35–39]. Frequency, mean and standard deviation was calculated. We also performed chi-square tests and Fisher’s exact test for categorical variables. We recategorized each barriers and sources of information into binary variables. Then we performed bivariable logistic regression analysis. The variables which were significant at *p* < 0.2 in our bivariable analysis were included in the multivariable logistic regression analysis which was used to identify factors associated with the perceived knowledge, attitude and practice level. The P-value of less than 0.05 was considered as statistically significant. Stata 16 (Stata Corp LLC, College Station, TX, USA) was used for data coding and analysis.

### Ethical approval

Ethical approval was obtained from the St. Luke’s International University Research Ethics Committee (approval number 20-E001). We obtained written informed consent from all the respondents after a complete disclosure of the research objectives, participation criteria, risks and benefits of participation, data sharing and privacy information.

## Results

A total of 542 responses were received via the QuestionPro software among them 132 participants were excluded due to their termination of the survey before responding to the questionnaire (filling time less than a minute) and missing information on their residency status. Similarly, we excluded six participants assuming that they were the inattentive responders as their answers to our question “In which planet do you live in?” was not correct. Hence, 404 respondents were analyzed in this study among which the majority of the respondents were between 26 to 45 years of age and almost two-third of them were male. The majority of respondents had completed up to the high school of education. Our study was dominated by Hindus and immigrants from Province 3 and Province 4 of Nepal consisted of more than 60% of our sample population. Even though we had respondents from almost all regions of Japan, the survey was predominantly respondents from the Kanto region (65.01%). Those with their residency status as dependents (26.73%) were highest among our respondents followed by students (23.76%), cooks (17.33%), business professionals (15.35%) and others including permanent residents and highly skilled laborers. About 55% of respondents had more than five years of stay in Japan. In the meantime, more than 50% of the respondents mentioned that the social networking sites including Facebook, Twitter, YouTube and some smart phone applications like Viber and Line are a good medium for receiving information regarding disaster preparedness. However, almost 60% of respondents mentioned that they would prefer to have that information in Nepali language, and less than 3% preferred Japanese language for information seeking. In addition to that, majority of respondents perceived language as the biggest barrier in accessing the disaster preparedness knowledge, attitude and practice (39.28%) followed by information deficit (28.31%), not having enough time (24.37%) and work pressure (6.71%). Some other barriers reported by the respondents were related to low-risk perception and unavailability of training facilities in their native language. When we stratified knowledge, attitude and practice level of disaster preparedness using Bloom’s criteria we found that despite of having a positive attitude, more than 80% of respondents had poor knowledge and more than 95% had a poor practice of disaster preparedness. (Table 1).

**Table 1:** Socio-demographic characteristics of the respondents (*N* = 404)

	**Frequency**	**Percentage**
**Age group**		
19–25	62	15.35
26–35	160	39.6
36–45	129	31.93
46 +	53	13.12
**Sex**		
Male	253	62.62
Female	151	37.38
**Education**		
$\le$ SEE	73	18.10
+ 2 completed	182	45.16
$\ge$ Bachelors completed	148	36.72
**Religion**		
Hindu	330	82.29
Buddhist	59	14.71
Others	12	2.99
**Province in Nepal**		
Province 1	26	6.44
Province 2	34	8.42
Province 3	168	41.58
Province 4	88	21.78
Province 5	65	16.09
Province 6	18	4.46
Province 7	5	1.24
**Region in Japan**		
Hokkaido	5	1.24
Tohoku	13	3.23
Kanto	262	65.01
Chubu	38	9.43
Kansai	28	6.95
Chugoku	14	3.47
Shikoku	12	2.98
Kyushu	31	7.69
**Type of visa**		
Student	96	23.76
Cook	70	17.33
Business	62	15.35
Dependent	108	26.73
Others	68	16.83
**Period of stay in Japan (in years)**		
< 5	177	44.81
$\ge$ 5	218	55.19
**Work status**		
Working	377	93.55
Not working	26	6.45
**Marital status**		
Single/ Divorced	107	26.49
Married	297	73.51
**Number of people living together, mean (SD)**	2.12 (1.25)	NA
**Past experience of any natural disasters**		
Yes	115	28.47
No	289	71.53
**Language preferred to access information on disaster preparedness**		
Japanese	9	2.23
English	51	12.62
Nepali	242	59.90
Japanese and English	4	0.99
Japanese and Nepali	19	4.70
English and Nepali	71	17.57
Language doesn’t matter	8	1.98
**Source of information**^a^		
Newspaper	75	6.24
TV	149	12.40
Family and Friends	180	14.97
Cellphones	200	16.64
Facebook	330	27.45
Twitter	143	11.90
YouTube	125	10.40
**Knowledge level of disaster preparedness**		
Good	74	18.32
Poor	330	81.68
**Attitude level of disaster preparedness**		
Good	208	51.49
Poor	196	48.51
**Practice level of disaster preparedness**		
Good	16	3.96
Bad	388	96.04
**Barriers in accessing KAP of disaster preparedness**^a^		
Language barrier	240	39.28
Information deficit	173	28.31
Not having enough time	149	24.37
Work pressure	41	6.71
Others	8	1.31

Number of missing respondents = 1 for education, region in Japan and work status, 3 for religion, 9 for period of stay in Japan and number of people living together

*SEE* Secondary Education Examination (standard terminology used for final examination of 10^th^ grade of school in Nepal), + *2 completed* Equivalent to the degree of high school graduates, *KAP* Knowledge, Attitude and Practice, *TV* Television, *SNS* Social Networking Sites, *SD* Standard Deviation, *NA* Not available, ^a^ Results derived from multiple response question

**Fig. 1:**
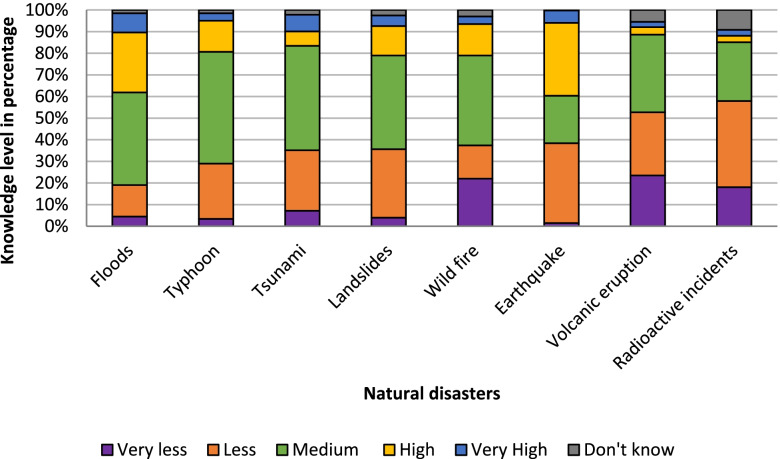
Knowledge level of Nepalese immigrants regarding various natural disasters

Furthermore, we observed that nearly 29% had been affected by some form of natural disasters in the past among which the majority reported being affected by an earthquake disaster either in Nepal or in Japan. Meanwhile, the majority of respondents had low to medium level of knowledge regarding each of the natural disasters; however, most had nearly 3 to 7 times higher knowledge on more frequent disasters like earthquakes, floods and typhoons compared to rare disasters like radioactive incidents or volcanic eruptions with a mean score of knowledge of 21.30 $\pm 5.75$ (mean $\pm SD$). (Fig. 1).

**Fig. 2:**
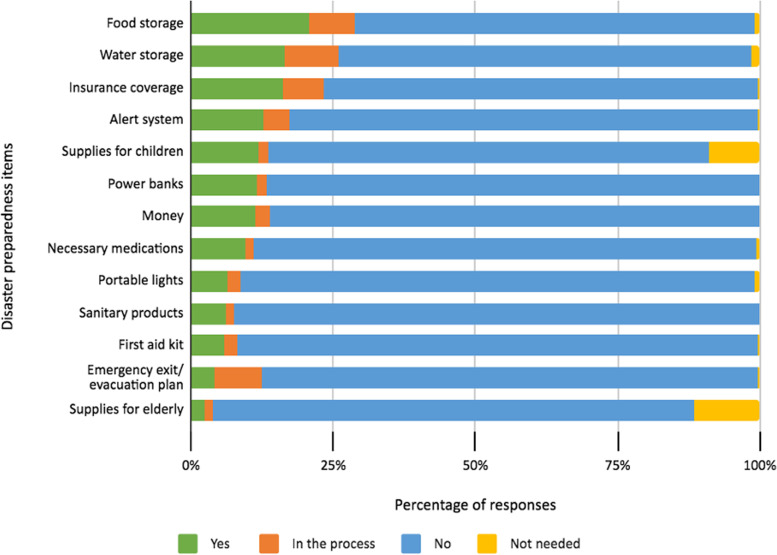
Disaster preparedness among Nepalese immigrants in Japan

On the contrary, we found that the respondents were concerned regarding every kind of natural disaster (mean $\pm SD$ = 29.12 $\pm$ 5.83). People were more concerned about typhoon (81.39%) followed by earthquakes (80.64%) and tsunamis (79.41%) and least concerned about wildfires (50.37%). Similarly, more than 70% of the respondents reported that somehow, they are not able to perform some preventive practices against natural disasters like collecting necessary information related to disasters, the evacuation shelters or preparing family emergency plan, survival kits, participating in awareness training, etc. Meanwhile, more than two-third of the respondents had not prepared even a few important items that are necessary to arrange before a natural disaster, such as water and food storage for at least three days, having an alert system to inform friends and relatives about their situation, preparing disaster kits including first aid kits, portable lights, necessary medications, some cash, power banks, sanitary products, supplies necessary for children or elderly, planning emergency evacuation and insurance coverage. We found that very few had already prepared the aforementioned items anticipating the risk of disasters and even fewer were in the process of preparing these items (mean $\pm SD$ = 15.86 $\pm$ 5.52). (Fig. 2).

We also stratified our sample population by their level of knowledge, attitude and practice of disaster preparedness and performed univariate analysis. Age, marital status, barriers, language preferred to access information on disaster preparedness and preferred source of information (namely newspaper, television, cellphone, YouTube and Twitter) were associated with the knowledge level of disaster preparedness. Whereas knowledge level itself and the language preferred to access information were significantly associated with the practice level. (Table 2).

**Table 2:** Proportion of knowledge, attitude and disaster preparedness practice among Nepali immigrants in Japan

	**Proportion**					
	**Poor knowledge**	**Good knowledge**	**Poor attitude**	**Good attitude**	**Bad practice**	**Good practice**
**Age group**						
19–25	0.14	0.22	0.16	0.15	0.15	0.12
26–35	0.39	0.41	0.40	0.39	0.40	0.31
36–45	0.32	0.34	0.32	0.32	0.31	0.44
46 +	0.15	0.04	0.12	0.14	0.13	0.13
***p*-value**	0.044*		0.882		0.773	
**Sex**						
Male	0.61	0.69	0.67	0.59	0.63	0.63
Female	0.39	0.37	0.33	0.41	0.37	0.37
***p*-value**	0.216		0.089		0.992	
**Education**						
$\le$ SEE	0.19	0.12	0.18	0.18	0.19	0.06
+ 2 completed	0.46	0.41	0.47	0.44	0.46	0.31
$\ge$ Bachelors completed	0.35	0.47	0.35	0.38	0.36	0.63
***p*-value**	0.115		0.771		0.081	
**Religion**						
Hindu	0.81	0.89	0.84	0.81	082	0.88
Non-Hindus	0.19	0.11	0.16	0.19	0.18	0.12
***p*-value**	0.085		0.381		0.578	
**Province in Nepal**						
Province 1	0.07	0.04	0.06	0.07	0.06	0.13
Province 2	0.08	0.08	0.11	0.06	0.09	0.00
Province 3	0.39	0.53	0.44	0.39	0.41	0.63
Province 4	0.23	0.15	0.17	0.26	0.22	0.06
Province 5	0.16	0.15	0.15	0.17	0.16	0.06
Province 6	0.05	0.04	0.05	0.04	0.04	0.06
Province 7	0.01	0.01	0.02	0.01	0.01	0.06
***p*-value**^**#**^	0.463		0.310		0.073	
**Region in Japan**						
Hokkaido	0.02	0.00	0.01	0.01	0.01	0.00
Tohoku	0.04	0.00	0.01	0.05	0.03	0.00
Kanto	0.65	0.64	0.68	0.62	0.65	0.63
Chubu	0.10	0.07	0.09	0.10	0.10	0.00
Kansai	0.07	0.07	0.06	0.08	007	0.00
Chugoku	0.03	0.05	0.04	0.03	0.04	0.00
Shikoku	0.03	0.04	0.02	0.04	0.03	0.12
Kyushu	0.06	0.14	0.10	0.06	0.07	0.25
***p*-value**^**#**^	0.186		0.179		0.074	
**Type of visa**						
Student	0.22	0.32	0.24	0.24	0.23	0.31
Cook	0.18	0.15	0.16	0.18	0.18	0.13
Business	0.17	0.07	0.16	0.14	0.16	0.06
Dependent	0.27	0.26	0.26	0.28	0.27	0.19
Others	0.16	0.20	0.18	0.16	0.16	0.31
***p*-value**	0.084		0.922		0.410	
**Period of stay in Japan (in years)**						
< 5	0.44	0.47	0.46	0.43	0.45	0.31
$\ge$ 5	0.56	0.53	0.54	0.57	0.55	0.69
***p*-value**	0.633		0.535		0.265	
**Work status**						
Working	0.95	0.86	0.94	0.93	0.94	0.81
Not working	0.05	0.14	0.06	0.07	0.06	0.19
***p*-value**^**#**^	0.015^**#**^		0.842		0.076	
**Marital status**						
Single/ Divorced	0.24	0.38	0.24	0.29	0.27	0.25
Married	0.76	0.62	0.76	0.71	0.73	0.75
***p*-value**	0.014*		0.268		0.891	
**Past exposure to natural disaster**						
Yes	0.28	0.28	0.30	0.27	0.28	0.31
No	0.72	0.72	0.70	0.73	0.72	0.69
***p*-value**	0.985		0.626		0.801	
**Language preferred to access KAP**						
At least some level of Japanese	0.06	0.16	0.07	0.09	0.07	0.19
English and/or Nepali	0.93	0.78	0.91	0.89	0.91	0.75
Language doesn’t matter	0.01	0.06	0.02	0.02	0.02	0.06
***p*-value**	0.001**		0.648		0.110	
**Barriers in accessing KAP of natural disaster preparedness**						
*Language barrier*						
Yes	0.60	0.42	0.44	0.56	0.43	0.50
No	0.40	0.58	0.42	0.58	0.57	0.50
***p*-value**	0.004**		0.603		0.568	
*Information deficit*						
Yes	0.44	0.38	0.39	0.47	0.43	0.38
No	0.56	0.62	0.61	0.53	0.57	0.62
***p*-value**	0.338		0.111		0.661	
*Not having enough time*						
Yes	0.36	0.42	0.40	0.34	0.37	0.31
No	0.64	0.58	0.60	0.66	0.63	0.69
***p*-value**	0.323		0.239		0.634	
*Work pressure*						
Yes	0.10	0.12	0.11	0.09	0.10	0.13
No	0.90	0.88	0.89	0.91	0.90	0.87
***p*-value**	0.526		0.487		0.751	
*Other barriers*						
Yes	0.02	0.04	0.01	0.03	0.02	0.00
No	0.98	0.96	0.99	0.97	0.98	0.10
***p*-value**^**#**^	0.165		0.286		1.000	
**Preferred source of information of natural disaster preparedness**						
*Newspaper*						
Yes	0.16	0.30	0.22	0.15	0.18	0.44
No	0.84	0.70	0.78	0.85	0.82	0.56
***p*-value**	0.006**		0.051		0.008**	
*Television*						
Yes	0.34	0.49	0.38	0.36	0.36	0.56
No	0.66	0.51	0.62	0.64	0.64	0.44
***p*-value**	0.020*		0.724		0.101	
*Family and relatives*						
Yes	0.45	0.45	0.41	0.48	0.45	0.38
No	0.55	0.55	0.59	0.52	0.55	0.62
***p*-value**	0.994		0.142		0.562	
*Cellphone*						
Yes	0.52	0.38	0.48	0.50	0.49	0.56
No	0.48	0.62	0.52	0.50	0.51	0.44
***p*-value**	0.026*		0.686		0.582	
*Facebook*						
Yes	0.80	0.89	0.83	0.80	0.82	0.81
No	0.20	0.11	0.17	0.20	0.18	0.19
***p*-value**	0.065		0.455		0.964	
*YouTube*						
Yes	0.28	0.43	0.36	0.26	0.30	0.56
No	0.72	0.57	0.64	0.74	0.70	0.44
***p*-value**	0.011*		0.026*		0.025*	
*Twitter*						
Yes	0.33	0.46	0.35	0.36	0.35	0.56
No	0.67	0.54	0.65	0.64	0.65	0.44
***p*-value**	0.036*		0.774		0.075	
**Perceived knowledge level**						
Poor knowledge	NA	NA	0.47	0.53	0.97	0.03
Good Knowledge	NA	NA	0.57	0.43	0.92	0.08
***p*-value**	NA		0.116		0.043*	

SEE = secondary education examination (standard terminology used for final examination of 10^th^ grade in Nepal), + 2 completed = equivalent to the degree of high school graduates, KAP = Knowledge, Attitude and Practice, *p*-value = obtained from chi-square test, *p*-value^**#**^ = *p*-value obtained from fisher’s exact test

NA = Not Available, the variable being the outcome variable for the chi-square test

^*^ = *p*-value < 0.05, ** = *p*-value < 0.01, ^**#**^ = *p*-value^**#**^ < 0.05

We also identified factors associated with the level of perceived knowledge, attitude and practice and found that age group above 46 years, religion, residency status, marital status, work status, language preferred to access information, preferred source of information (newspaper, television, cellphone, YouTube and Twitter) and language barrier were significantly associated with the knowledge level; however, only sex and source of information (YouTube) was found to be associated with the attitude level and source of information (Newspaper and YouTube) and the perceived knowledge level were significantly associated with practice level in our bivariable logistic regression analysis at *p* < 0.5. Similarly, our multivariable logistic regression analysis showed that those who preferred smartphone to seek information from had 54% less knowledge than those who don’t (aOR: 0.46, 95% CI = (0.26 – 0.82)). Also, those who identified Japanese language as one of the barrier in accessing the knowledge, attitude and practice had significantly lower knowledge than those who don’t perceive Japanese language as a barrier. We also observed that those who prefer YouTube as a source of information had 43% poor attitude than those who don’t. However, no other factors were significantly associated with the perceived knowledge, attitude and practice level of disaster preparedness after adjusting for all possible covariates under analysis. We also included the interaction between knowledge, attitude and practice in our model however the interaction was not significant at *p* < 0.05 hence, we showed the model without interaction in this study. (Table 3).

**Table 3:** Bivariable and multivariable logistic regression model on knowledge, attitude and practice of Nepalese immigrants regarding disaster preparedness

	**Crude Odds ratio (95% CI) (*N* = 404)**			**Adjusted Odds ratio (95% CI) (*N* = 403)**		
	**Poor knowledge**	**Poor attitude**	**Poor practice**	**Poor knowledge**	**Poor attitude**	**Poor practice**
**Age group**						
19–25	1	1	1	1	1	1
26–35	0.66 (0.33 – 1.33)	1.02 (0.57 – 1.84)	0.97 (0.18 – 5.12)	0.63 (0.24 – 1.64)	1.30 (0.61 – 2.79)	0.61 (0.07 – 4.95)
36–45	0.69 (0.34 – 1.41)	1.05 (0.57 – 1.92)	1.72 (0.35 – 8.54)	0.83 (0.28 – 2.52)	1.37 (0.57 – 3.31)	1.78 (0.16 – 19.80)
≥ 46	0.17 (0.05 – 0.63) **	1.30 (0.62 – 2.72)	1.18 (0.16 – 8.65)	0.25 (0.05 – 1.19)	2.01 (0.74 – 5.46)	1.86 (0.12 – 28.10)
**Type of visa**						
Students	1	1	1	1	1	1
Cook	0.56 (0.25 – 1.23)	1.13 (0.61 – 2.11)	0.53 (0.10 – 2.84)	1.47 (0.49 – 4.38)	1.36 (0.59 – 3.11)	0.34 (0.04 – 3.30)
Business	0.26 (0.09 – 0.73) *	0.90 (0.47 – 1.70)	0.30 (0.03 – 2.62)	0.48 (0.13 – 1.70)	1.01 (0.43 – 2.40)	0.13 (0.01 – 1.83)
Dependents	0.64 (0.32 – 1.26)	1.11 (0.64 – 1.93)	0.52 (0.12 – 2.24)	1.14 (0.48 – 2.78)	1.40 (0.69 – 2.84)	0.37 (0.05 – 2.81)
Others	0.85 (0.41 – 1.77)	0.90 (0.48 – 1.68)	1.44 (0.40 – 5.20)	0.80 (0.30 – 2.13)	1.23 (0.55 – 2.72)	0.54 (0.09 – 3.13)
**Marital status**						
Single/ Divorced	1	1	1	1	1	1
Married	0.52 (0.30 – 0.88) *	0.78 (0.50 – 1.21)	1.08 (0.34 – 3.44)	0.78 (0.33 – 1.86)	0.54 (0.27 – 1.08)	2.43 (0.41 – 14.6)
**Work status**						
Working	1	1	1	1	1	1
Non-working	3.06 (1.33 – 7.04) **	1.10 (0.49 – 2.44)	3.65 (0.97 – 13.73)	2.01 (0.74 – 5.48)	1.20 (0.49 – 2.91)	3.25 (0.65 – 16.21)
**Language preference**						
Some Japanese	1	1	1	1	1	1
Nepali and/or English	0.31 (0.15 – 0.68)**	0.71 (0.34 – 1.47)	0.33 (0.09 – 1.230	0.53 (0.21 – 1.32)	0.54 (0.23 – 1.22)	0.71 (0.14 – 3.64)
Language doesn’t matter	1.67 (0.35 – 7.93)	0.68 (0.14 – 3.24)	1.38 (0.12 – 15.36)	2.01 (0.35 – 11.64)	0.52 (0.10 – 2.66)	1.80 (0.12 – 27.12)
**Preferred source of information**						
* Newspaper*						
No	1	1	1	1	1	1
Yes	2.21 (1.24 – 3.94)**	0.61 (0.36 – 1.00)	3.66 (1.32 – 10.17)*	1.58 (0.74 – 3.39)	0.56 (0.30 – 1.04)	2.09 (0.54 – 8.09)
*Television*						
No	1	1	1	1	1	1
Yes	1.82 (1.09 – 3.03)*	0.93 (0.62 – 1.39)	2.28 (0.83 – 6.25)	1.22 (0.68 – 2.39)	1.28 (0.78 – 2.100	1.04 (0.27 – 4.03)
* Smartphone*						
No	1	1	1	1	1	1
Yes	0.56 (0.33 – 0.94)*	1.08 (0.73 – 1.60)	1.33 (0.48 – 3.63)	0.46 (0.26 – 0.82)**	1.13 (0.75 – 1.71)	1.37 (0.45 – 4.16)
* Twitter*						
No	1	1	1	1	1	1
Yes	1.94 (1.16 – 3.26)*	0.62 (0.40 – 0.94)*	3.01 (1.10- 8.29)*	1.63 (0.91 – 2.90)	1.20 (0.78 – 1.85)	2.23 (0.70 – 7.10)
* YouTube*						
No	1	1	1	1	1	1
Yes	1.72 (1.03 – 2.87)*	1.06 (0.71 – 1.60)	2.44 (0.89 – 6.69)	1.49 (0.82 – 2.72)	0.57 (0.36 – 0.92)*	2.19 (0.369– 6.98)
**Language barrier to access KAP**						
No	1	1	1	1	1	1
Yes	2.11 (1.26 – 3.51) **	0.90 (0.61 – 1.33)	1.34 (0.49 – 3.64)	0.54 (0.30 – 0.97)*	1.10 (0.72 – 1.69)	1.10 (0.35 – 3.49)

Multivariable model adjusted for age, type of visa, marital status, work status, language preference, preferred source of information and barriers to access KAP

*N* Number of sample size included in the analysis, *KAP* Knowledge, Attitude and Practice, *CI* Confidence interval

^*^ = *p*-value < 0.05, ** = *p*-value < 0.01

## Discussion

This study assessed the perceived knowledge, attitude and practice and explored the barriers regarding natural disaster preparedness among Nepalese immigrants residing in Japan. The results showed that the Nepalese immigrants have a low level of knowledge and practice with slightly a positive attitude about disaster preparedness. Several factors such as sex, area of residence, educational status and the residency status were associated with the knowledge, attitude and practice of individuals regarding natural disaster preparedness. We identified Japanese language as the major barrier in assessing the knowledge and practice on disaster preparedness followed by difficulties in seeking health information.

The observed knowledge level of Nepalese immigrants on disaster preparedness was substantially lower compared to Japanese natives [40]. However, most respondents had higher knowledge about more frequent disasters compared to rare disasters like volcanic eruptions and radioactive incidents which might reflect on the effect of past exposure to the disaster situations and the associated knowledge, attitude and practice [41, 42]. Meanwhile, a study by Tam G. et al. identified that more than half of the respondents in their study from Japan had good household disaster preparedness which was 12 times higher than that of the existing practice of Nepalese immigrants [43]. A household survey conducted in China also showed almost similar results as in Japan where the respondents had more than 50% awareness rate of knowledge regarding disaster preparedness [44]. This indicates that there is a considerable disparity in assessing knowledge and practice regarding disaster preparedness among the Japanese population as well as among immigrants living in Japan. In addition, higher knowledge on disaster preparedness has been associated with higher level of preparedness practice which was also evident in our study; however, the difference was far less than studies conducted in other parts of the world [45].

Several studies in the past have identified community engagement as a crucial factor in reducing disaster damage and enhancing disaster preparedness. However, we identified that most Nepalese do not participate in community drills because of the language barrier [46, 47]. The health-seeking behavior of Nepalese immigrants residing in other developed countries, such as the United Kingdom (UK), was higher than those residing in Japan. This suggests a need for a certain level of intervention in enhancing the health care seeking behavior of Nepalese immigrants residing in Japan as this is the key factor in improving knowledge of immigrants regarding various diseased conditions or events like natural disasters [48]. In addition to this, Japanese language has always been perceived as a major barrier in accessing health care by Nepalese immigrants. Some prefectural government had started multilingual counselling facilities; however, these are very limited in number. Hence, Japan government should provide better translation facilities to the immigrant population [49].

This study has several strengths and limitations. The findings of this study might be useful in establishing disaster preparedness programs for Nepalese immigrants which could be utilized for other tribes of immigrant populations residing in Japan with similar backgrounds. Similarly, this study can work as a strong appeal to the Japanese government in restructuring their government websites where people can seek the required information. On the other hand, the study findings may not be generalizable to all Nepalese immigrant communities residing in Japan as the participants were recruited from the social media. In contempt of these limitations, the present study evaluates the knowledge, attitude and practice of Nepalese immigrants regarding disaster preparedness and shows existing pitfalls of Japan government in helping vulnerable groups of the community to prepare themselves for the worst disaster outcomes.

Overall, the results suggest the necessity for establishing knowledge sharing platforms such as providing disaster awareness materials in the Nepali language on relevant government websites and fostering the system of disaster drills and practices in the occupational setting or in educational institutions. The Japanese government should place more emphasis on disaster preparedness awareness programs for international immigrants according to their needs.

## Conclusion

This study observed that Nepalese immigrants residing in Japan had a very low knowledge and practice regarding disaster preparedness and several other factors such as language barrier and insufficient information available on the Japanese government website were identified as added concerns. Thus, the availability of language translation services in every health care sector, also in the government offices of Japan might bring substantial change in the health information seeking behavior of the immigrant population residing in Japan, including Nepalese. Meanwhile, authorities should focus on the most popular source of information among Nepalese immigrants like Facebook in order to disseminate their information to the majority of people. There is need for policy reform and development of guidelines focusing especially on the considerable Nepali community residing in Japan to connect all these dots that exist in health care accessibility and seeking information.

## Supplementary Information

**Additional file 1.**

## Data Availability

The questionnaire used in this study is provided as a supplementary material in this manuscript. The datasets used and/or analyzed during the current study are available from the corresponding author on reasonable request.

## Abbreviations

IFRCS: International Federation of Red Cross Society
EED: Emergency Events Database
GDP: Gross Domestic Product
US: United States
USD: United States Dollar
NRNA: Non-Residential Nepalese Association
CVR: Content Validity Ratio
FGD: Focus Group Discussion
CVI: Content Validity Index
I-CVI: Item level Content Validity Index
S-CVI: Scale level Content Validity Index
IRB: International Review Board
UK: United Kingdom,
SEE: Secondary Education Examination
aOR: Adjusted Odds Ratio
CI: Confidence Interval
SNS: Social Networking Sites
