# Supplementary material for: Knowledge, attitude, practice and perceived barriers of natural disaster preparedness among Nepalese immigrants residing in Japan
Source: BMC Public Health. 2022 Mar 12;22:492. doi: 10.1186/s12889-022-12844-3 (PMC8918343; doi:10.1186/s12889-022-12844-3)
Supplement: Supplementary file 1 — Additional file 1. [file 12889_2022_12844_MOESM1_ESM.pdf]

Knowledge, Attitude, Practice and Perceived Barriers of Natural Disaster Preparedness Among Nepalese Immigrants Residing in Japan

Aliza K C Bhandari [1*], Osamu Takahashi[1]

[1]St. Luke's International University, Graduate School of Public Health, Tokyo, Japan

***Corresponding author**

18mp204@slcn.ac.jp/20dp001@slcn.ac.jp

Survey Questionnaire: English version

*The Nepali version of this questionnaire would be provided only on reasonable request

A. Disaster preparedness knowledge and practice related questions

1. Are you aware about natural disaster? (Click on anyone option)
   Yes
   No

2. Have you or your family ever been affected by a natural disaster? (Click on anyone option)
   Yes
   No
   If yes, please specify the type of natural disaster …………………………………

3. Do you live in an area which is highly populated/ heavily crowded? (Click on anyone option)
   Yes
   No

4. What is the likelihood of the following natural disasters to affect the area you live in Japan in the upcoming year? (Click on appropriate options)

	**1 very low chance**	**2 low chance**	**3 medium chance**	**4 high chance**	**5 very high chance**	**6/0 Don't know / no answer**
Earthquake						
Volcanic eruption						
Floods						
Landslides						
Typhoon						
Wild fire						
Tsunami						
Radioactive incidents						

5. How much knowledge do you have regarding the disaster preparedness of following natural disasters? (Click on appropriate options)

	**1 Very less**	**2 Less**	**3 medium**	**4 High**	**5 Very High**	**6/0 Don't know**
Earthquake						
Volcanic eruption						
Floods						
Landslides						
Typhoon						
Wild fire						
Tsunami						
Radioactive incidents						

6. How concerned are you with natural disaster preparedness knowledge you have about the following disasters? (Click on appropriate options)

	**1 Not concerned at all**	**2 Not concerned**	**3 medium concerned**	**4 Concerned**	**5 Highly concerned**	**6/0 Don't know**
Earthquake						
Volcanic eruption						
Floods						
Landslides						
Typhoon						
Wild fire						
Tsunami						
Radioactive incidents						

7. Have you prepared yourself for any natural disaster mentioned in question number 6 that might occur in the future? (Click on appropriate options)

	**0 Don't want to do**	**1 Cannot do**	**2 Have not done**	**3 Planning to do**	**4 Have already done**
Collected the information related to natural disaster management					
Conversation with disaster management representative					
Prepared a family emergency plan					
Participated in preparing a neighborhood emergency plan					
Prepared disaster survival kits					
Collected information on disaster shelter area of one's locality					
Collected information on the route to the disaster shelter area of one's locality					
Taken special training on disaster management					
Trained other members in the community for disaster management					
Signed up with entities that provides emergency news or alert systems in English or Nepali language					

8. Have you arranged for the following in case of an emergency or a natural disaster? (Click on appropriate options)

**S.N.**		**1 No**	**2 In the process**	**3 Yes**	**0 Not needed**
1.	A minimum of 72 hours of water supply or enough water storage for at least two days.				
2.	A minimum of 72 hours of food supply				
3.	An alert system for family and community like miking, phone calls, SMS, etc.)				
4.	Disaster kits or emergency bag that contains the following items:				
i.	Portable flashlights or other lights sources				
ii.	First aid kit containing gauze pieces, cotton, antiseptic solutions, alcohol swabs, bandages, etc.)				
iii.	Necessary hygiene and sanitation products (hand wash soap, sanitary pads, tissues, hand sanitizer, toothbrush, toothpaste, towel)				
iv.	Long lasting battery cell phones with power banks				
v.	Necessary medications				
vi.	Money				
vii.	Important supplies for children (like milk formula, napkins, warm clothes, etc.)				
viii.	Important supplies for aged or people with special needs (like wheelchair, hearing/visual aids, medication, etc.)				
5.	Emergency exit and evacuation plan				
6.	Insurance coverage				

9. In your opinion, how will the community receive information about a natural disaster or an emergency situation? (Click on all that applies)

- ☐ Newspapers
- ☐ Television
- ☐ Family and friends
- ☐ Cellphones
- ☐ Facebook
- ☐ Twitter
- ☐ YouTube
- ☐ Other internet sources please specify ……..)
- ☐ Others (please specify …………………………………………………)

10. Have you participated in natural disaster preparedness or training or management programs in Japan?
    Yes
    No
    If yes, please specify the source (Click on all that applies)

- [ ] Government/ Ward office/ prefecture
- [ ] Non-government organizations
- [ ] Nepal embassy/ NRNA
- [ ] Others (please specify ……………………………………………………)

11. In which language do you prefer to receive information about a natural disaster or an emergency situation? (Click any one option)

- [ ] Japanese
- [ ] English
- [ ] Nepali
- [ ] Japanese and English
- [ ] Japanese and Nepali
- [ ] English and Nepali
- [ ] Language doesn't matter

B. Barriers related question

1. What are the perceived barriers in accessing the knowledge regarding natural disaster preparedness? (Click on all that applies)

- [ ] Language
- [ ] Information deficit
- [ ] Don't have enough time
- [ ] Work pressure
- [ ] Others **(Please specify and write freely what you feel is the barrier for you to not get enough information on natural disaster preparedness)**

C. Socio-demographic characteristics related questions

1. Age (in numbers) ………………………..
2. Sex

i. Male
ii. Female
iii. Others

3. Highest education level
   i. Below 10th grade
   ii. 10th grade
   iii. +2/ High school completed
   iv. Bachelors completed
   v. Masters completed and/or above
4. Religion
   i. Hindu
   ii. Buddhist
   iii. Christian
   iv. Muslim
   v. Others
5. Province in Nepal
   i. Province 1
   ii. Province 2
   iii. Province 3
   iv. Province 4
   v. Province 5
   vi. Province 6
   vii. Province 7
6. Prefecture in Japan
   1"Hokkaidō" 2"Aomori" 3"Iwate" 4"Miyagi" 5"Akita" 6"Yamagata" 7"Fukushima" 8"Ibaraki" 9"Tochigi" 10"Gunma" 11"Saitama" 12"Chiba" 13"Tōkyō" 14"Kanagawa" 15"Niigata" 16"Toyama" 17"Ishikawa" 18"Fukui" 19"Yamanashi" 20"Nagano" 21"Gifu" 22"Shizuoka" 23"Aichi" 24"Mie" 25"Shiga" 26"Kyōto" 27"Ōsaka" 28"Hyōgo" 29"Nara" 30"Wakayama" 31"Tottori" 32"Shimane" 33"Okayama" 34"Hiroshima" 35"Yamaguchi" 36"Tokushima" 37"Kagawa" 38"Ehime" 39"Kōchi" 40"Fukuoka" 41"Saga" 42"Nagasaki" 43"Kumamoto" 44"Ōita" 45"Miyazaki" 46"Kagoshima" 47"Okinawa"
7. City in Japan (in word) …………………………………………………..
8. Type of residency status or visa status
   i. Student
   ii. Cook
   iii. Business
   iv. Dependent
   v. Others (please specify ……………………)
9. Period of stay in Japan (please write in completed years)
   …………………………………….
10. Do you have an income generating work in Japan?
    I. Yes
    II. No
11. Time spent at work on average per week in hours……………………
12. Marital status
    I. Unmarried/ Single
    II. Married
    III. Divorced
13. Number of people or family members staying together in Japan …………………
